# Real-Time Clinical Decision Support Based on Recurrent Neural Networks for In-Hospital Acute Kidney Injury: External Validation and Model Interpretation

**DOI:** 10.2196/24120

**Published:** 2021-04-16

**Authors:** Kipyo Kim, Hyeonsik Yang, Jinyeong Yi, Hyung-Eun Son, Ji-Young Ryu, Yong Chul Kim, Jong Cheol Jeong, Ho Jun Chin, Ki Young Na, Dong-Wan Chae, Seung Seok Han, Sejoong Kim

**Affiliations:** 1 Division of Nephrology and Hypertension, Department of Internal Medicine Inha University College of Medicine Incheon Republic of Korea; 2 Department of Computer Science and Engineering Seoul National University Seoul Republic of Korea; 3 Department of Health Science and Technology, Graduate School of Convergence Science and Technology Seoul National University Seoul Republic of Korea; 4 Department of Internal Medicine Seoul National University Bundang Hospital Seongnam Republic of Korea; 5 Department of Internal Medicine Seoul National University Hospital Seoul Republic of Korea; 6 Center for Artificial Intelligence in Healthcare Seoul National University Bundang Hospital Seongnam Republic of Korea

**Keywords:** acute kidney injury, recurrent neural network, prediction model, external validation, internal validation, kidney, neural networks

## Abstract

**Background:**

Acute kidney injury (AKI) is commonly encountered in clinical practice and is associated with poor patient outcomes and increased health care costs. Despite it posing significant challenges for clinicians, effective measures for AKI prediction and prevention are lacking. Previously published AKI prediction models mostly have a simple design without external validation. Furthermore, little is known about the process of linking model output and clinical decisions due to the black-box nature of neural network models.

**Objective:**

We aimed to present an externally validated recurrent neural network (RNN)–based continuous prediction model for in-hospital AKI and show applicable model interpretations in relation to clinical decision support.

**Methods:**

Study populations were all patients aged 18 years or older who were hospitalized for more than 48 hours between 2013 and 2017 in 2 tertiary hospitals in Korea (Seoul National University Bundang Hospital and Seoul National University Hospital). All demographic data, laboratory values, vital signs, and clinical conditions of patients were obtained from electronic health records of each hospital. We developed 2-stage hierarchical prediction models (model 1 and model 2) using RNN algorithms. The outcome variable for model 1 was the occurrence of AKI within 7 days from the present. Model 2 predicted the future trajectory of creatinine values up to 72 hours. The performance of each developed model was evaluated using the internal and external validation data sets. For the explainability of our models, different model-agnostic interpretation methods were used, including Shapley Additive Explanations, partial dependence plots, individual conditional expectation, and accumulated local effects plots.

**Results:**

We included 69,081 patients in the training, 7675 in the internal validation, and 72,352 in the external validation cohorts for model development after excluding cases with missing data and those with an estimated glomerular filtration rate less than 15 mL/min/1.73 m2 or end-stage kidney disease. Model 1 predicted any AKI development with an area under the receiver operating characteristic curve (AUC) of 0.88 (internal validation) and 0.84 (external validation), and stage 2 or higher AKI development with an AUC of 0.93 (internal validation) and 0.90 (external validation). Model 2 predicted the future creatinine values within 3 days with mean-squared errors of 0.04-0.09 for patients with higher risks of AKI and 0.03-0.08 for those with lower risks. Based on the developed models, we showed AKI probability according to feature values in total patients and each individual with partial dependence, accumulated local effects, and individual conditional expectation plots. We also estimated the effects of feature modifications such as nephrotoxic drug discontinuation on future creatinine levels.

**Conclusions:**

We developed and externally validated a continuous AKI prediction model using RNN algorithms. Our model could provide real-time assessment of future AKI occurrences and individualized risk factors for AKI in general inpatient cohorts; thus, we suggest approaches to support clinical decisions based on prediction models for in-hospital AKI.

## Introduction

Acute kidney injury (AKI) is a common clinical condition that can be attributed to multiple causes in various clinical settings. AKI increases mortality, morbidity, length of hospital stay, and health care costs [1-4]. According to the National Confidential Enquiry on Patient Outcomes and Death reports, approximately 17% of AKI is estimated to be avoidable and preventable [5]. Therefore, many research efforts have been expended to detect AKI early on and to manage patients with high risk [6]. Nevertheless, the reported incidence of AKI is 17%-25% in the hospital setting [7,8] and it has continued to rise globally during the recent decades [9]. Markedly increasing amounts of electronic health record (EHR) data and recent developments in machine learning techniques offer greater possibilities for the improvement of quality of care and medical research [10]. Various clinical decision support systems that use EHR and machine learning have been increasingly reported for various diseases [11-14]. Machine learning methods can incorporate tremendously large number of features in the model compared with conventional regression models and thus enable the use of nonlinear algorithms. As a result, in AKI research, several studies have adopted machine learning methods such as random forest and neural network models and reported improved model performance [15-17]. Major risk factors associated with in-hospital AKI include the use of various nephrotoxins, repeatedly measured laboratory findings, and vital signs that are dynamic rather than static variables [18-20]. The recurrent neural network (RNN) is a powerful tool used to handle such sequential data [21]; various RNN models have shown excellent performance in the field of natural language processing and time-series forecasting models [22,23]. Although the RNN model is a promising approach, time-updated predictive models for AKI using the RNN algorithm are still in their infancy [24]. Only a few studies have investigated these, and they have not been externally validated [20,25]. Moreover, despite their enhanced performance, these neural network models cannot provide insights into how to link clinical decision supports; therefore, interpreting the output using these models can be difficult. In this respect, neural network models have been criticized as being black-box models [26]. Previous studies on clinical decision support for AKI have not implemented predictions for AKI development but have only served as alarm systems for the timely diagnosis of AKI according to diagnostic criteria [27,28]. Therefore, in this study, we propose an externally validated RNN-based prediction model for in-hospital AKI and aimed to provide a framework to link the developed model with clinical decision support.

## Methods

### Study Population

This study was performed in accordance with the recommendations laid out in the World Medical Association Declaration of Helsinki. The study protocol was approved by the Institutional Review Boards (IRBs) of the Seoul National University Bundang Hospital (SNUBH; IRB No. B-1912/583-406) and Seoul National University Hospital (SNUH; IRB No. H-1911-043-1076). Written consent was waived by the IRB because of the retrospective nature of the study, and all data were completely anonymized. Study populations comprised all patients aged 18 years or older who were hospitalized for more than 48 hours at the SNUBH from 2013 to 2017 (training and internal validation cohorts) and at the SNUH from 2013 to 2017 (external validation cohort). These 2 tertiary hospitals are affiliated with each other. However, they are located in different regions of Korea and have different patient populations and EHR systems. The exclusion criteria were as follows: (1) no baseline or follow-up creatinine (Cr) measurements, (2) baseline estimated glomerular filtration rate (eGFR) less than 15 mL/min/1.73 m^2^ or Cr greater than 4.0 mg/dL or end-stage kidney disease at admission, (3) no other laboratory test results used in the model, (4) no BMI or vital sign measurements, and (5) an AKI diagnosis at admission (day 1).

### Data Collection

All demographics, laboratory values, vital signs, and clinical conditions were obtained from the EHR of each hospital. The features that are considered as risk factors of AKI from the related literature or are correlated with AKI development were selected for model development [18]. A total of 107 variables were included in the model. These are summarized in Multimedia Appendix 1. Each variable was classified as either static or dynamic. The static variables were assigned to time-invariant values during hospitalization, and the dynamic variables were assigned to values that were updated on a daily basis. Demographics and comorbidities were static variables, while laboratory tests, vital signs, and clinical conditions were dynamic variables. The use of medications during admission was incorporated in the model as a dynamic categorical variable, and medication history before admission was treated as a static variable. Patient comorbidities were classified into 17 categories according to the International Statistical Classification of Disease and Related Health Problems (10th revision) codes from which the Charlson Comorbidity Index was assessed [29]. BMI was calculated from the height and weight measured at admission. Laboratory values included Cr, white blood cells, hemoglobin, platelets, albumin, sodium, potassium, chloride, aspartate aminotransferase, alanine aminotransferase, blood urea nitrogen, total CO_2_, bilirubin, calcium, glucose, creatine kinase, lipase, and troponin I. The means of laboratory values that were measured more than once each day were used in our model. Medications included well-known nephrotoxic agents, such as nonsteroidal anti-inflammatory drugs, aminoglycoside, vancomycin, and colistin. Vital signs included systolic, diastolic, and mean arterial blood pressures; pulse; and body temperature, which were usually measured 3 times a day in general wards. Therefore, the mean, maximum, and minimum values of the vital signs during a day were used as different variables.

### AKI and Baseline Creatinine Definitions

AKI was defined according to the Kidney Disease: Improving Global Outcomes (KDIGO) Clinical Practice Guideline for AKI [30]. Because urine volume data were not available, AKI stage was defined based on serum Cr levels. Baseline Cr levels were determined by searching the minimum serum Cr level within a period of 2 weeks before admission. If there were no serum Cr measurements during this period, the minimum value of Cr measured within 90 or 180 days before admission was used as the baseline Cr. In the absence of Cr measurements up to 180 days before admission, the serum Cr value measured on the first day of hospitalization was defined as the baseline Cr.

### Data Preprocessing and Statistical Analysis

**Figure 1:**
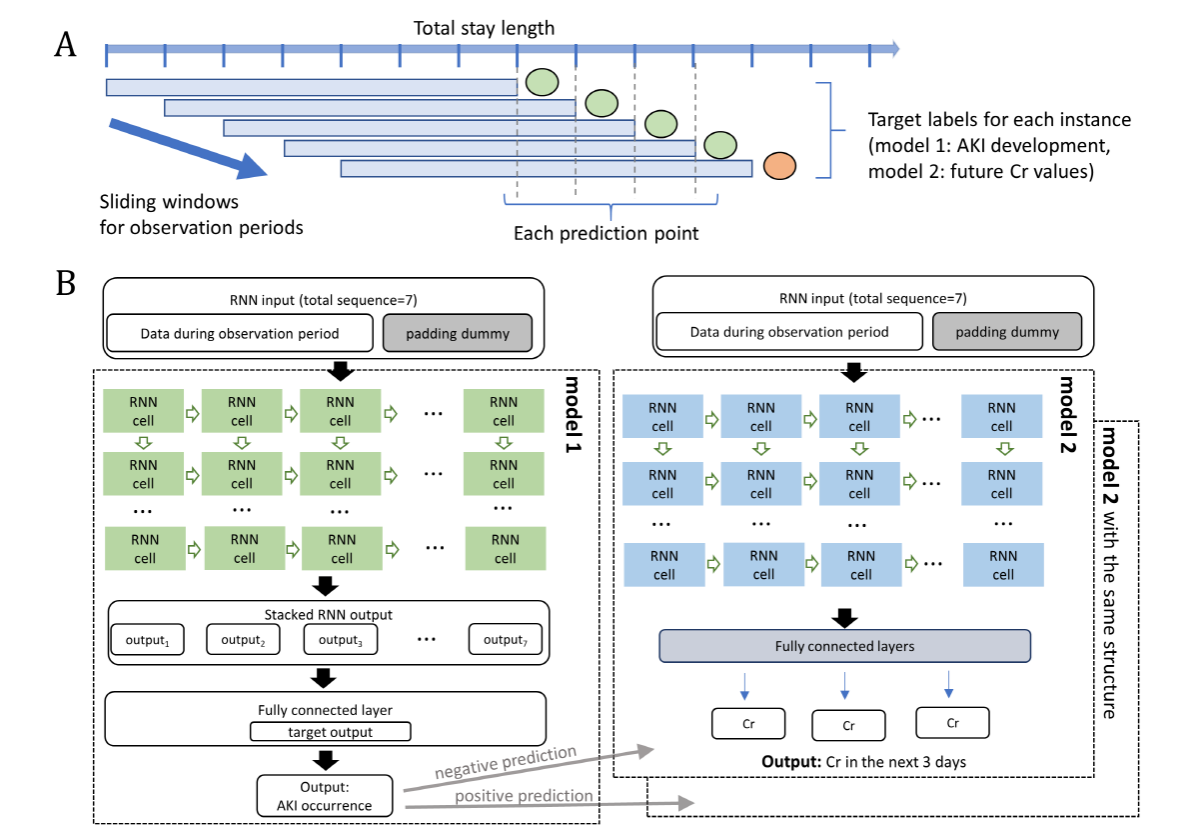
Architectural overview of the developed model using the recurrent neural network (RNN). (A) The sliding window approach for handling sequential data and (B) the 2-stage hierarchical model comprising models 1 and 2. Model 1 could predict the occurrence of acute kidney injury (AKI) in the next 7 days from the present, and model 2 could provide the predicted value of serum creatinine (Cr) for the next 3 days.

During data collection, error values were treated as missing, and outliers of all variables were examined and removed after review by domain experts. Patients with missing variables at baseline were excluded according to the exclusion criteria. The last observation carried forward method was used for missing values after baseline. Variables were scaled using the min–max normalization before training the neural network. For continuous prediction of AKI, the training and validation data sets were organized as multiple sliding windows of features and target labels fed to the input layer of the RNN model (Figure 1A). The length of the sliding window was selected as 7 days and features up to 2 weeks after admission were utilized. Therefore, all time points were considered for both patients with AKI and non-AKI during the length of stay before AKI occurrence or discharge. The SNUBH data set was divided into a training set (69,081/76,756, 90%) and an internal validation set (7675/76,756, 10%) using the stratified random split. Categorical variables are expressed as numbers and percentages, and continuous variables as means (SD). The chi-square test and *t* test were used to compare differences in baseline characteristics between the training and validation cohorts. *P* values <.05 were considered statistically significant.

### Model Development and Assessment

We developed 2 different models (models 1 and 2) with stacked RNN algorithms. Model 1 had a many-to-one architecture with 7 sequential inputs and 1 prediction output. The outcome variable for model 1 was the occurrence of AKI in the next 7 days. Padding and masking techniques were used for instances with sequences shorter than the length of the sliding window. The padded sequences were not used in training and inference processes. To compare the performance with model 1, we developed an additional gradient boosting model based on the same training data set. The gradient boosting model was trained using the XGBoost algorithm. In model 2, we constructed a prediction model of the trajectory of Cr values after 24, 48, and 72 hours with available Cr values during the observation window. Model 2 had a many-to-many structure with an output length of 3 on 7 input sequences. To improve the predictive accuracy of model 2, we developed a 2-stage hierarchical RNN prediction model. That is, based on the results of model 1, different types of model 2 were applied. These are the models for each patient group that are or are not predicted to have AKI. The architectures of models 1 and 2 are illustrated in Figure 1. The optimal hyperparameters of each model were determined using a fivefold cross validation. The tuned hyperparameters include number of hidden RNN neurons, number of hidden layers, dropout, activation functions, and batch size in the stacked RNN model, and learning rate, depth of a tree, and minimum child weight in the XGBoost model. Cross entropy loss function and the AdamW optimizer were used to train the RNN models. The entire data set was imbalanced due to the relatively low incidence of in-hospital AKI. Therefore, we applied a class weight parameter to the loss function to handle class imbalances. Alpha dropout, L2 regularization, and early stopping approaches were implemented to prevent overfitting. Batch normalization was applied to each RNN layer for efficient and effective learning. The learning rate was set to 10^–4^ for pretraining, then to 10^–6^ for early stop learning. In the process of model development, different sample sizes were tested to evaluate the sensitivity of the developed models. As shown in Table 1 in the *Results* section, the overall distribution of features is quite different between the training and external validation data sets. Therefore, we additionally fine-tuned our models to overcome the heterogeneity of independent data sets. Specifically, we refitted our model using a small proportion of data (7234/72,352, 10%) from the external data set. The refitted model was validated with the rest of the external data set (65,118/72,352, 90%). Model performances were assessed based on the area under the receiver operating characteristic curve (AUC), accuracy, sensitivity, specificity, positive predictive value, negative predictive value, and F1 score for model 1 and based on the mean-squared error (MSE) for model 2.

### Model Explainability for Clinical Decision Support

The neural network algorithm cannot directly offer any explanations regarding the clinical meaning of features. Therefore, to identify the association between multiple features and response (the occurrence of AKI), we examined the following approaches using model-agnostic methods in model 1: (1) global interpretation with Shapley Additive Explanations (SHAP), partial dependence plots (PDPs), and accumulated local effects plots; and (2) instance-wise interpretation with individual conditional expectation (ICE) plots. The SHAP method is based on Shapley values from game theory [31]. Shapley values indicate marginal contribution of a feature to the difference between the actual prediction and the mean prediction for all possible coalitions of features [32]. The global feature importance was presented by SHAP feature importance plots. PDP provides average marginal predictions across all instances when the feature of interest is forced to be a certain value [33]. Similarly, ICE plots indicate an average marginal effect of a feature for individual instances. PDP and ICE plots can intuitively show the relationship between specific features and the outcome variable [34]. In model 2, we applied ICE to predict the future Cr values, whereby the model output was estimated from new instances with modified feature values as well. Given that PDP is not reliable when the features are highly correlated, we also presented accumulated local effects plots. In an accumulated local effects plot, feature values are divided by set intervals and the differences in the prediction between the upper and lower bounds of the interval are calculated [33]. The estimated differences are accumulated, and the mean prediction is centered at 0.

## Results

### Study Population

A total of 482,467 patients (182,976 in the SNUBH and 299,491 in the SNUH) were initially screened from the EHR data obtained from each participating hospital. After considering the exclusion criteria, 69,081 patients were finally included in the SNUBH training data set, 7675 in the SNUBH internal validation data set, and 72,352 in the SNUH external validation data set (Multimedia Appendix 2). The characteristics of the study population are listed in Table 1. Patients in the training data set were older than those in the external validation data set (59.8 versus 57.1 years). The mean baseline eGFR was higher in the training data set than in the external validation data set (94.9 versus 89.4 mL/min/1.73 m^2^). Although more patients had hypertension or diabetes in the training data set, the mean Charlson Comorbidity Index was higher in the external validation data set. There was no difference in baseline characteristics between the training and internal validation data sets. During the 2-week period after admission, the cumulative incidence of AKI (any stage) was 5.91% in the training data set and 3.63% in the external validation data set (Multimedia Appendix 3). The cumulative incidence of severe AKI (stages 2 or 3) was 1.58% and 1.11% in the training and external validation data sets, respectively.

**Table 1:** Baseline characteristics of the training and validation data sets.

Variables			SNUBH^a^ training set (n=69,081)		SNUBH validation set (n=7675)		SNUH^b^ validation set (n=72,352)		*P* value^c^
Age (years), mean (SD)			59.8 (16.5)		59.6 (16.6)		57.1 (16.0)		<.001
Male sex, n (%)			36,732 (53.2)		4114 (53.6)		37,405 (51.7)		<.001
Body mass index (kg/m^2^), mean (SD)			23.8 (3.5)		23.8 (3.5)		23.2 (3.5)		<.001
Stay length (days), mean (SD)			8.7 (14.2)		8.5 (10.4)		6.9 (11.7)		<.001
ICU^d^ admission, n (%)			4478 (6.5)		475 (6.2)		2340 (3.2)		<.001
Charlson Comorbidity Index, mean (SD)			1.0 (1.4)		1.1 (1.4)		1.1 (1.4)		<.001
**Specific preexisting comorbidities**									
	Hypertension, n (%)	9455 (13.7)		995 (13.0)		6624 (9.2)		<.001	
	Diabetes mellitus, n (%)	7187 (10.4)		769 (10.0)		6513 (9.0)		<.001	
	Ischemic heart disease, n (%)	7452 (10.8)		790 (10.3)		6289 (8.7)		<.001	
	Heart failure, n (%)	1500 (2.2)		169 (2.2)		793 (1.1)		<.001	
Baseline eGFR^e^ (mL/min/1.73 m^2^), mean (SD)			94.9 (38.9)		94.6 (37.4)		89.4 (21.9)		<.001
No CKD^f^ or CKD 1 or 2 (≥60 mL/min/1.73 m^2^), n (%)			60,201 (87.1)		6683 (87.1)		65,310 (90.3)		<.001
CKD G3a (45-59 mL/min/1.73 m^2^), n (%)			4839 (7.0)		550 (7.2)		4188 (5.8)		
CKD G3b (30-44 mL/min/1.73 m^2^), n (%)			2629 (3.8)		279 (3.6)		1940 (2.7)		
CKD G4 (15-29 mL/min/1.73 m^2^), n (%)			1412 (2.0)		163 (2.1)		914 (1.3)		
Hemoglobin (g/dL), mean (SD)			13.0 (2.1)		13.0 (2.1)		13.0 (2.0)		.54
Albumin (g/dL), mean (SD)			4.0 (0.6)		4.0 (0.6)		4.1 (0.5)		<.001
Bilirubin (mg/dL), mean (SD)			0.9 (1.5)		0.9 (1.6)		0.9 (1.5)		.03
Calcium (mg/dL), mean (SD)			8.8 (0.6)		8.8 (0.6)		9.1 (0.6)		<.001
Glucose (mg/dL), mean (SD)			126.4 (55.5)		125.8 (54.1)		121.1 (48.9)		<.001
Sodium (mEq/L), mean (SD)			138.9 (3.8)		138.8 (3.8)		139.8 (3.3)		<.001
Potassium (mEq/L), mean (SD)			4.1 (0.4)		4.2 (0.5)		4.2 (0.4)		<.001
Chloride (mEq/L), mean (SD)			102.5 (4.0)		102.5 (4.0)		103.5 (3.5)		<.001
AST^g^ (IU/L), mean (SD)			47.3 (245.3)		46.7 (213.2)		32.4 (108.7)		<.001
ALT^h^ (IU/L), mean (SD)			42.6 (192.1)		42.0 (154.1)		30.5 (100.4)		<.001
Total CO_2_ (mEq/L), mean (SD)			24.7 (3.3)		24.7 (3.3)		25.6 (3.6)		<.001
Platelet (10^3^/μL), mean (SD)			232.9 (85.0)		232.3 (86.7)		235.8 (479.1)		<.001
White blood cell count (cells/mm^2^), mean (SD)			8.2 (7.1)		8.3 (8.3)		7.3 (5.5)		.10
**Medication use**									
	RAS^i^ blockers, n (%)	5494 (8.0)		610 (7.9)		6039 (8.3)		.007	
	Diuretics, n (%)	3437 (5.0)		381 (5.0)		3723 (5.1)		.14	
	NSAIDs^j^, n (%)	16,974 (24.6)		1874 (24.4)		13,290 (18.4)		<.001	
Systolic blood pressure (mmHg), mean (SD)			128.7 (16.5)		128.6 (16.5)		124.8 (15.8)		<.001
Diastolic blood pressure (mmHg), mean (SD)			74.1 (10.8)		74.0 (10.7)		76.2 (10.7)		<.001
Heart rate (beats/minute), mean (SD)			78.6 (14.4)		78.7 (14.3)		77.7 (14.2)		<.001
Body temperature (°C), mean (SD)			36.7 (0.5)		36.7 (0.5)		36.5 (0.4)		<.001

^a^SNUBH: Seoul National University Bundang Hospital.

^b^SNUH: Seoul National University Hospital.

^c^*P* value between the SNUBH training set and the SNUH validation set (variables are not statistically different between the SNUBH training set and the SNUBH validation set).

^d^ICU: intensive care unit.

^e^eGFR: estimated glomerular filtration rate.

^f^CKD: chronic kidney disease.

^g^AST: aspartate aminotransferase.

^h^ALT: alanine aminotransferase.

^i^RAS: renin–angiotensin system.

^j^NSAIDs: nonsteroidal anti-inflammatory drugs.

### Prediction of AKI Development

**Figure 2:**
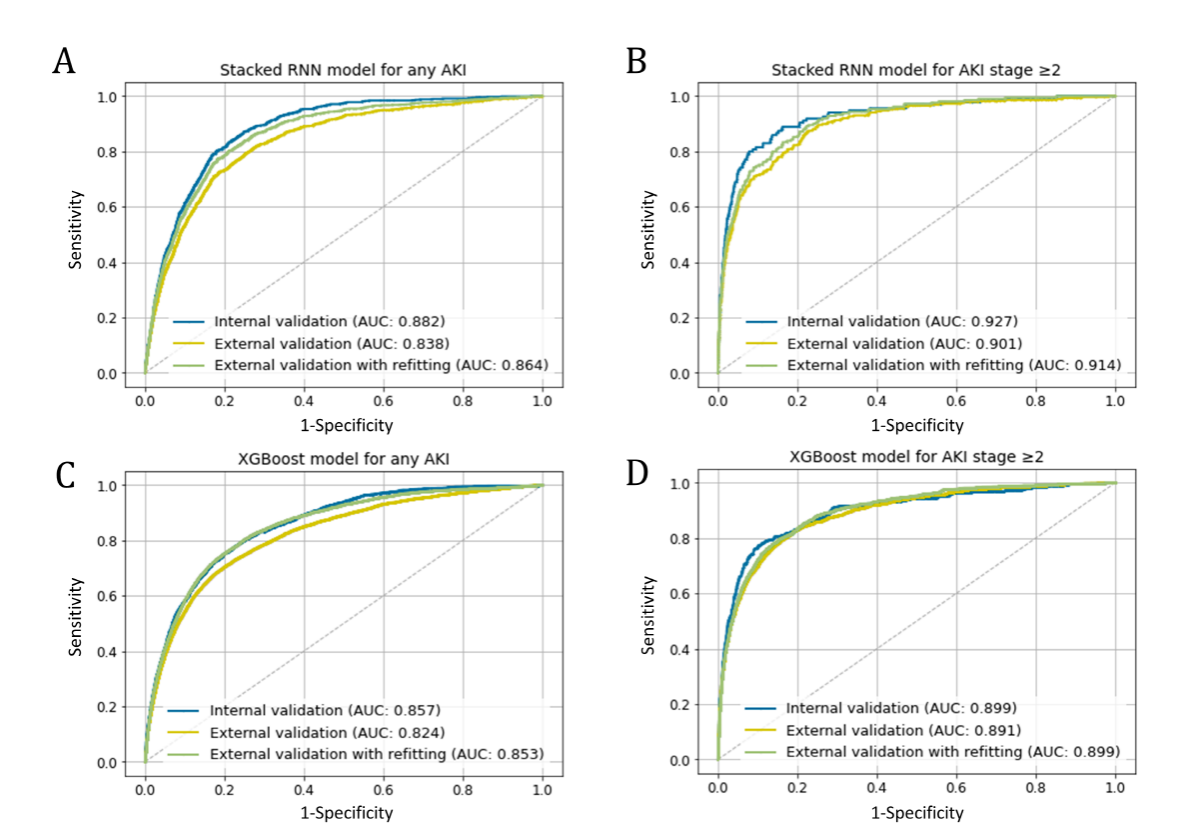
The receiver operating characteristic curve for model 1. (A) The stacked RNN model for any AKI, (B) the stacked RNN model for AKI stage 2 or higher, (C) the XGBoost model for any AKI, and (D) the XGBoost model for AKI stage 2 or higher. AKI: acute kidney injury; RNN: recurrent neural network.

First, we assessed the performance of model 1 to predict AKI development (any stage and stage 2 or higher) within the next 7 days based on different algorithms. Overall, the AUC was higher in the stacked RNN model than in the XGBoost model for any AKI and stage 2 or higher AKI. The AUC of the RNN model was 0.88/0.84 (internal/external validation) for any AKI and 0.93/0.90 (internal/external validation) for stage 2 or higher AKI, while the AUC of the XGBoost model was 0.86/0.82 (internal/external validation) for any AKI and 0.90/0.89 (internal/external validation) for stage 2 or higher AKI (Figure 2). Overall, better performance was found with the larger training sample size, which might be due to the class imbalance of the data set, suggesting that 80%-90% of the split ratio was sufficient in training (Multimedia Appendix 4). The model performance on the external validation set was slightly lower than that on the internal validation set. However, the performance of the updated model was improved, reducing the difference in the AUC between the internal and external validation sets. The RNN model to predict stage 2 or higher AKI revealed the highest performance. The evaluation metrics other than AUC are shown for different probability cutoff values in Multimedia Appendices 5 and 6. We evaluated model 2 based on model 1 for any AKI, considering the AKI incidence rate.

### Prediction of Creatinine Trajectory

We constructed and validated the prediction model (model 2) for Cr trajectory separately in the positive and negative prediction groups based on the prediction results of model 1 for any AKI. The MSE values are presented in Table 2, and ranged from 0.03 to 0.06 in the internal validation case, and from 0.06 to 0.09 in the external validation case. The refitted model in the external validation data set showed the improved MSE of 0.03-0.08. Overall, the MSE values at different prediction points (24, 48, and 72 hours) were comparable to each other.

**Table 2:** Predictive performance of model 2 for the future value of serum creatinine (Cr).

Subgroup and validation method			MSE at different time points						
			24 hours		48 hours		72 hours		
**Positive prediction**									
	Internal validation	0.04		0.04		0.06			
	External validation	0.06		0.06		0.09			
	External validation (refitting)	0.05		0.06		0.08			
**Negative prediction**									
	Internal validation	0.03		0.04		0.04			
	External validation	0.06		0.06		0.08			
	External validation (refitting)	0.03		0.05		0.05			

### Application of Interpretability Techniques on the Developed Models

**Figure 3:**
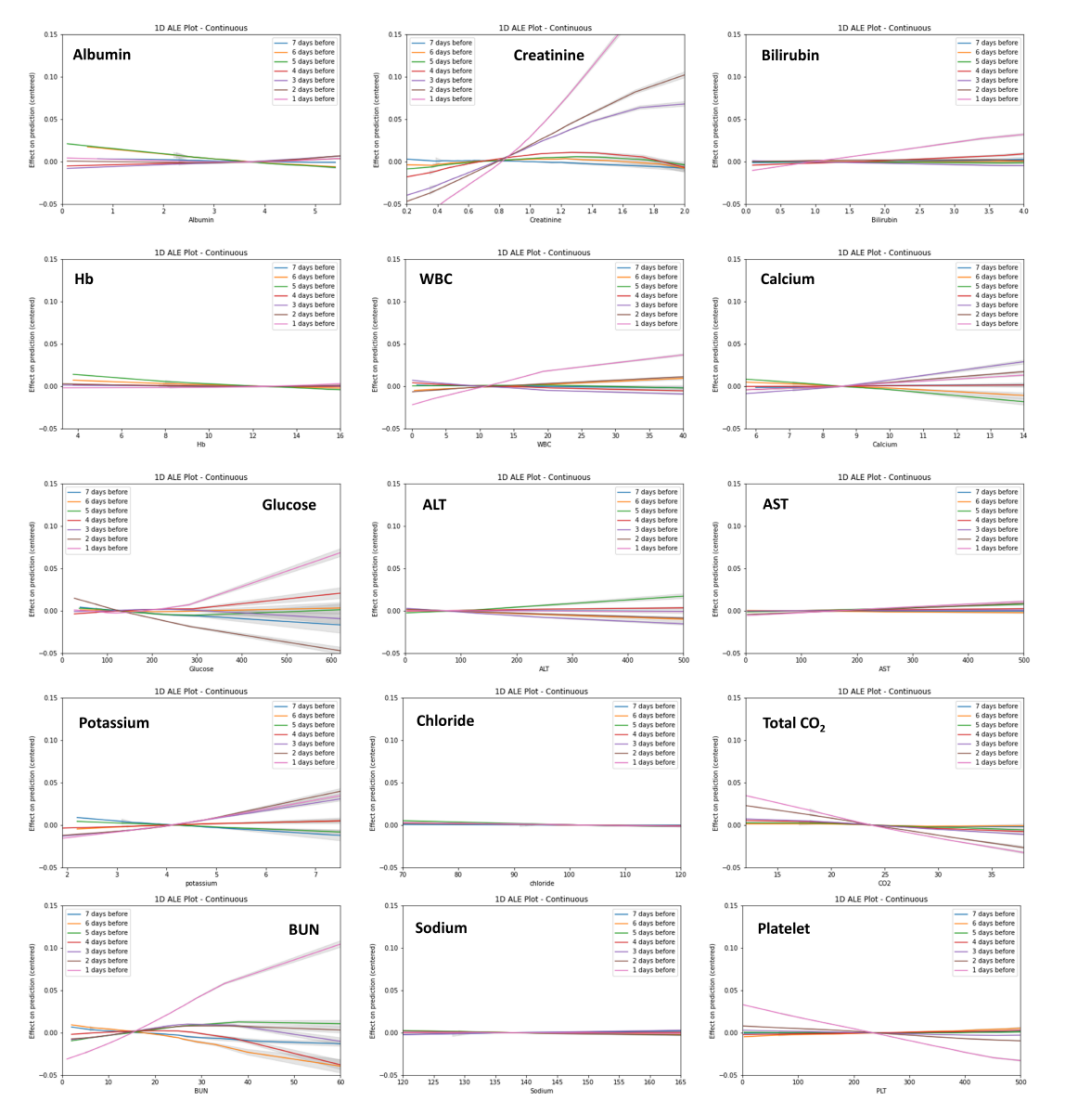
Accumulated local effects plots for the selected variables at the different time gaps from the prediction point. The same y-scale was used for all plots. AST: aspartate aminotransferase; ALT: alanine aminotransferase; BUN: blood urea nitrogen; Hb: hemoglobin; WBC: white blood cell count.

**Figure 4:**
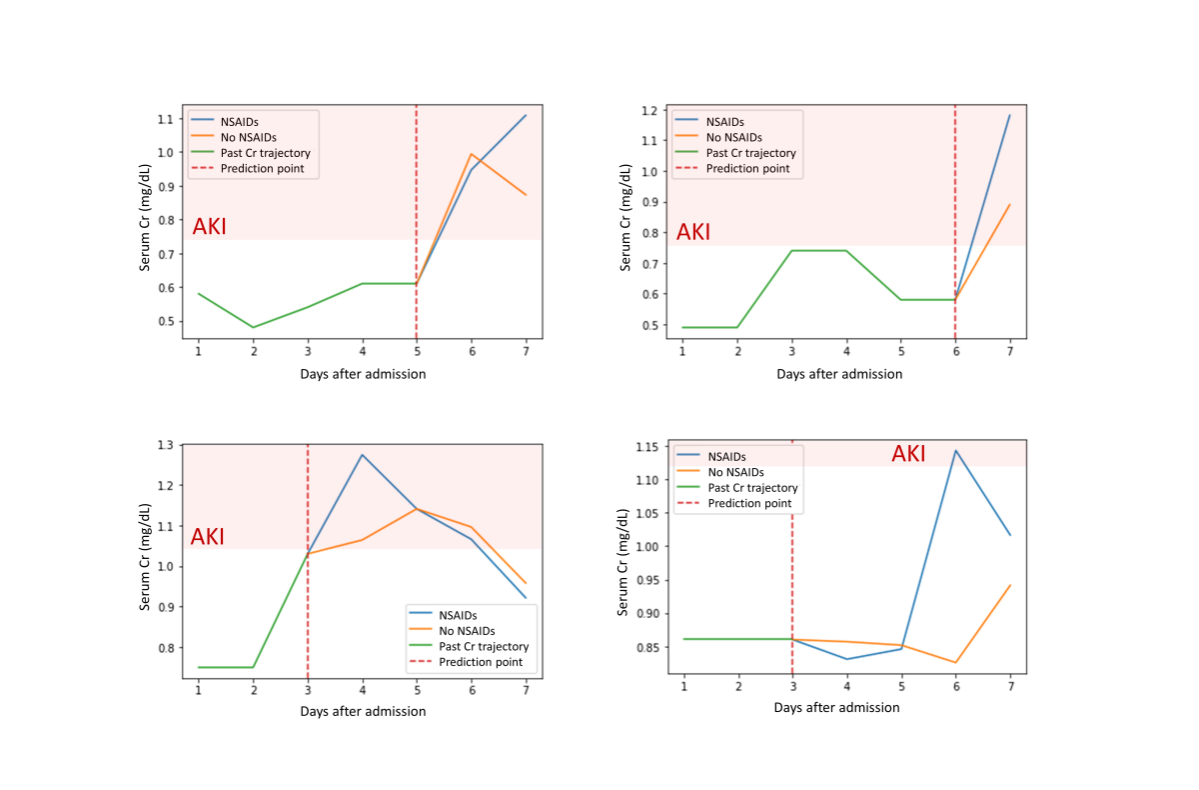
Individual interpretation with individual conditional expectations (green lines: the past Cr values; blue lines: predicted Cr values on certain nephrotoxic drugs; orange lines: predicted Cr value when nephrotoxic drugs are discontinued; and dotted red lines: prediction point). Modified drugs are NSAIDs in these 4 cases. AKI: acute kidney injury; Cr: creatinine; NSAIDs: nonsteroidal anti-inflammatory drugs.

Application of model 1 allows the identification of patients with high AKI risk. Moreover, the future values of Cr can be predicted using model 2. However, risk assessment and predicted laboratory values are insufficient for the management of patients with high risk. The prediction model itself could not provide measures on how to manage or prevent AKI. First, we examined feature importance obtained by mean SHA*P* values in model 1 (Multimedia Appendix 7). Baseline eGFR and serum Cr were the top 2 features in both RNN and XGBoost models. The remaining features showed relatively less impact on prediction. To determine the feature importance in the RNN model, the SHA*P* values were also averaged over the sequence length (7 days); thus, many static variables were of high rank. However, the importance ranking of some dynamic features, such as pulse rate, diuretic use, and white blood cell count, was found to increase over time (Multimedia Appendix 8). To illustrate the relationships between the AKI risk and each feature, we have presented PDP and ICE plots in Multimedia Appendix 9, where yellow lines denote the average probability of AKI versus the selected feature values in the studied patients (PDP), and each black line represents an individual’s probability (ICE plot). These plots provide global and instance-level model explanations. They primarily offer visual representations of a selected feature’s effect on AKI probability. Overall, substantial changes in AKI probability with the level of features were observed, especially for aspartate aminotransferase, platelet count, white blood cell count, and vital signs (eg, blood pressure and pulse rate). The patterns of ICE plots were quite different in individual patients. However, PDP could not fully explain the feature–response relationship in each patient. We did not find a clear effect of some clinically meaningful features such as hemoglobin and albumin on AKI prediction. However, accumulated local effects plots better showed the association between these features and AKI prediction (Figure 3). Therefore, we performed an additional analysis using accumulated local effects plots to evaluate the feature effects at different time gaps from the prediction point (Figure 3 and Multimedia Appendix 10). Vital signs, white blood cell counts, and Cr levels had the greatest effect the day before the prediction point, while albumin and hemoglobin showed the greatest effect 5-6 days before the prediction point. Some dynamic features, such as the use of medication, could be considered as correctable features in actual, clinical settings. In this regard, we estimated the predicted trajectories of serum Cr values according to the use of nephrotoxic drugs. Predicted values of Cr with and without nephrotoxic drug administration are shown in Figure 4. The presented cases represent the past Cr values (green lines), predicted values (blue lines), and predicted values after the discontinuation of nephrotoxic drugs (orange lines). These approaches could help clinicians make decisions regarding the prevention and intervention for future AKI occurrence.

## Discussion

### Principal Findings

In this study, we developed a continuous prediction model for in-hospital AKI using the RNN algorithm with external validation and demonstrated its applicability for the support of clinical decision making. The developed model was constructed for all general inpatients based on the use of various dynamic and static clinical features, showing relatively good performance. External validation was performed with data from an independent center. Furthermore, we showed examples relevant to the presentation of feature information to help actual clinical decisions at the global and individual patient level using several model-agnostic interpretation methods.

To prevent and manage AKI more effectively, timely diagnosis and intervention are emphasized. Thus, AKI is an area of interest for the application of predictive clinical models. To date, numerous studies have been published on predictive models related to AKI [18]. The identification and appropriate management of patients with high risk could improve patient outcomes and reduce economic burden in health care facilities [35]. Nevertheless, previous studies of the AKI prediction model have mainly focused on specific population groups, such as patients who are critically ill or those who underwent cardiovascular surgery, thus making generalization difficult [36-38]. Moreover, most models have not been externally validated, and there are only few models that are capable of real-time assessments. In a recent report of artificial intelligence research, only 6% of conducted studies performed external validation [39]. Many predictive models used clinical or laboratory data collected at the time of admission or at specific time points, such as during the preoperative period or admission [40-42]. However, the definition of AKI is dynamic and includes the concept of time duration (Cr increase by at least 0.3 mg/dL in 48 hours or 1.5 times over 1 week) as shown in the KDIGO definition [30]. Not only the changes in Cr levels, but also the changed rates or slopes were important in clinical decisions [43]. In this regard, the RNN algorithms could have a considerable strength and could fully utilize the sequential information of feature values. The RNN models were found to be effective in learning sequential data and demonstrated superior model performance over conventional models developed using data at one time point [44-46]. These reflect the trends of clinical variables and are expected to be advantageous for real-time risk assessment.

In this study, we developed 2-stage hierarchical prediction models (model 1 and model 2) using the stacked RNN structure. Model 1 focused on the distinction of patients with high risk of AKI from those without risk. Model 1 using stacked RNN layers predicted any AKI with an AUC of 0.84-0.88 and stage 2 or higher AKI with an AUC of 0.90-0.93. In particular, the RNN model outperformed the XGBoost-based model for both any AKI and stage 2 or higher AKI. Gradient boosting algorithm has been frequently utilized for the model of AKI prediction in recent related studies. The overall performance of the gradient boosting models was reported to have an AUC of 0.67-0.77 for AKI stage 1 or higher and an AUC of 0.84-0.87 for AKI stage 2 or higher in the next 48 hours [47-49]. Model 2 intended to predict the future values of serum Cr that could help physicians understand and interpret the models in a more intuitive manner. We adapted a study design that is updated on a daily basis because most laboratory tests or medications usually do not change more than once a day in the general ward of institutions. Similarly, to minimize the burden of real-time data processing, a daily AKI alert system was implemented into the EHR, which demonstrated its effectiveness for the diagnosis of unlooked AKI [28]. In this study, the overall model performance was somewhat different between the internal and external validation cohorts. It may be attributed to different patient characteristics, such as age and comorbidities, and to the patterns of clinical practice in the 2 centers. Specifically, the incidence of AKI stage 1 was quite different between the 2 centers. In this regard, some authors suggest multiple external validations for a generalized prediction model [50]. The construction of a generalizable prediction model in superpopulations is burdensome in terms of time and cost. Therefore, we additionally fine-tuned our model to overcome the heterogeneity of independent data sets. This approach has been employed in recent related studies [32,49]. Although only a small amount of data was used for refitting, the model performance was greatly improved. The results of the refitted models were comparable to those of the internal validation data set.

However, machine learning–based models do not explain why their decisions are right, and it is uncertain how they relate the results to clinical decision making. To apply the prediction results in clinical practice, clinicians must understand how the risk assessment is derived. Unlike conventional regression models, neural network models process enormous amounts of input data through complex, multiple hidden layers and weight parameters that make it difficult for clinicians to understand and interpret the network structure. In this context, machine learning models are usually referred to as black boxes. Therefore, we presented some model-agnostic interpretation approaches, such as the SHAP feature importance, PDP with ICE plots, and accumulated local effects plots. These methods are practical and helpful in utilizing neural network–based models and could help us identify correctable risk factors at the individual patient level. ICE plots could offer insights into how selected features affect prediction results in the black-box model. As shown in ICE plots, quite different relationships between feature values and prediction outcomes were observed in each patient. This suggests interindividual heterogeneity of feature contributions. PDP does not reflect the heterogeneous effects between individuals and requires an assumption of independence [33]. In this regard, the accumulated local effects plot provides a better interpretation of the association between features and predictions. Accumulated local effects plots are unbiased and superior to the PDP when evaluating feature effects in data sets with correlated features. Nevertheless, the interpretation methods themselves do not indicate causal inferences between features and the model output. Currently, various feature engineering techniques are actively being studied and constitute promising fields of artificial intelligence. Therefore, human-friendly interpretable models that reflect possible causal inferences will be developed in the future.

### Limitations

There are some limitations associated with this study. First, we used retrospective data in model training and validations. Thus, our results do not indicate the performance of the model in actual clinical practice. Second, because the model is updated daily, its application is more appropriate for patients in general wards than for those who are critically ill. Nevertheless, we developed an AKI prediction model with a relatively high performance and validated it externally. We presented the model interpretation methods for the RNN model based on sequential data and showed an example of the effective utilization of an AKI prediction model.

### Conclusion

Our study demonstrated how to support clinical decisions based on RNN-based prediction models in the clinical setting. Our model can provide real-time assessment of future AKI occurrences and individualized risk factors for AKI in general inpatient cohorts.

## Appendix

Multimedia Appendix 1 List of variables used in model development.

Multimedia Appendix 2 Flow diagram for study participants.

Multimedia Appendix 3 Cumulative incidence of AKI.

Multimedia Appendix 4 The AUC of the developed model according to different training sample sizes.

Multimedia Appendix 5 Evaluation metrics of model 1 for different probability cutoffs (any stage AKI).

Multimedia Appendix 6 Evaluation metrics of model 1 for different probability cutoffs (AKI stage ≥2).

Multimedia Appendix 7 SHAP feature importance plot for model 1.

Multimedia Appendix 8 SHAP feature importance plots according to different time points.

Multimedia Appendix 9 Partial dependence plots and individual conditional expectation plots.

Multimedia Appendix 10 Accumulated local effects plots for the vital sign variables at different time gaps from the prediction point.

Multimedia Appendix 11 CONSORT-EHEALTH checklist (V 1.6.1).

## Abbreviations

AKI: acute kidney injury
AUC: area under the receiver operating characteristic curve
DBP: diastolic blood pressure
EHR: electronic health record
eGFR: estimated glomerular filtration rate
ICE: individual conditional expectation
ICU: intensive care unit
PDP: partial dependence plots
RAS: renin–angiotensin system
RNN: recurrent neural network
SBP: systolic blood pressure
SHAP: Shapley Additive Explanations
WBC: white blood cell count

## References

[1] Chertow GM, Burdick E, Honour M, Bonventre JV, Bates DW (2005). Acute kidney injury, mortality, length of stay, and costs in hospitalized patients. J Am Soc Nephrol.

[2] Liangos O, Wald R, O'Bell JW, Price L, Pereira BJ, Jaber BL (2006). Epidemiology and outcomes of acute renal failure in hospitalized patients: a national survey. Clin J Am Soc Nephrol.

[3] Wang HE, Muntner P, Chertow GM, Warnock DG (2012). Acute kidney injury and mortality in hospitalized patients. Am J Nephrol.

[4] Coca SG, Peixoto AJ, Garg AX, Krumholz HM, Parikh CR (2007). The prognostic importance of a small acute decrement in kidney function in hospitalized patients: a systematic review and meta-analysis. Am J Kidney Dis.

[5] Stewart J, National Confidential Enquiry into Patient Outcome and Death (2009). Adding insult to injury: A review of the care of patients who died in hospital with a primary diagnosis of acute kidney injury (acute renal failure).

[6] Vanmassenhove J, Kielstein J, Jörres A, Biesen WV (2017). Management of patients at risk of acute kidney injury. Lancet.

[7] Susantitaphong P, Cruz DN, Cerda J, Abulfaraj M, Alqahtani F, Koulouridis I, Jaber BL, Acute Kidney Injury Advisory Group of the American Society of Nephrology (2013). World incidence of AKI: a meta-analysis. Clin J Am Soc Nephrol.

[8] Zeng X, McMahon GM, Brunelli SM, Bates DW, Waikar SS (2014). Incidence, outcomes, and comparisons across definitions of AKI in hospitalized individuals. Clin J Am Soc Nephrol.

[9] Lameire NH, Bagga A, Cruz D, De Maeseneer J, Endre Z, Kellum JA, Liu KD, Mehta RL, Pannu N, Van Biesen W, Vanholder R (2013). Acute kidney injury: an increasing global concern. Lancet.

[10] Sutherland S, Goldstein S, Bagshaw S (2018). Acute Kidney Injury and Big Data. Contrib Nephrol.

[11] Wang F, Preininger A (2019). AI in Health: State of the Art, Challenges, and Future Directions. Yearb Med Inform.

[12] Medic G, Kosaner Kließ M, Atallah L, Weichert J, Panda S, Postma M, El-Kerdi A (2019). Evidence-based Clinical Decision Support Systems for the prediction and detection of three disease states in critical care: A systematic literature review. F1000Res.

[13] Chen J, Chokshi S, Hegde R, Gonzalez J, Iturrate E, Aphinyanaphongs Y, Mann D (2020). Development, Implementation, and Evaluation of a Personalized Machine Learning Algorithm for Clinical Decision Support: Case Study With Shingles Vaccination. J Med Internet Res.

[14] Contreras I, Vehi J (2018). Artificial Intelligence for Diabetes Management and Decision Support: Literature Review. J Med Internet Res.

[15] Chiofolo C, Chbat N, Ghosh E, Eshelman L, Kashani K (2019). Automated Continuous Acute Kidney Injury Prediction and Surveillance: A Random Forest Model. Mayo Clin Proc.

[16] Tomašev N, Glorot X, Rae JW, Zielinski M, Askham H, Saraiva A, Mottram A, Meyer C, Ravuri S, Protsyuk I, Connell A, Hughes CO, Karthikesalingam A, Cornebise J, Montgomery H, Rees G, Laing C, Baker CR, Peterson K, Reeves R, Hassabis D, King D, Suleyman M, Back T, Nielson C, Ledsam JR, Mohamed S (2019). A clinically applicable approach to continuous prediction of future acute kidney injury. Nature.

[17] Simonov M, Ugwuowo U, Moreira E, Yamamoto Y, Biswas A, Martin M, Testani J, Wilson FP (2019). A simple real-time model for predicting acute kidney injury in hospitalized patients in the US: A descriptive modeling study. PLoS Med.

[18] Hodgson LE, Sarnowski A, Roderick PJ, Dimitrov BD, Venn RM, Forni LG (2017). Systematic review of prognostic prediction models for acute kidney injury (AKI) in general hospital populations. BMJ Open.

[19] Wang Y, Fang Y, Teng J, Ding X (2016). Acute Kidney Injury Epidemiology: From Recognition to Intervention. Contrib Nephrol.

[20] Meyer A, Zverinski D, Pfahringer B, Kempfert J, Kuehne T, Sündermann SH, Stamm C, Hofmann T, Falk V, Eickhoff C (2018). Machine learning for real-time prediction of complications in critical care: a retrospective study. Lancet Respir Med.

[21] Karako K, Chen Y, Tang W (2019). On medical application of neural networks trained with various types of data. Biosci Trends.

[22] Hewamalage H, Bergmeir C, Bandara K (2021). Recurrent Neural Networks for Time Series Forecasting: Current status and future directions. International Journal of Forecasting.

[23] Goldberg Y (2017). Neural network methods for natural language processing.

[24] Wilson FP (2020). Machine Learning to Predict Acute Kidney Injury. Am J Kidney Dis.

[25] Tran N, Sen S, Palmieri T, Lima K, Falwell S, Wajda J, Rashidi Hooman H (2019). Artificial intelligence and machine learning for predicting acute kidney injury in severely burned patients: A proof of concept. Burns.

[26] Castelvecchi D (2016). Can we open the black box of AI?. Nature.

[27] Wilson FP, Greenberg JH (2018). Acute Kidney Injury in Real Time: Prediction, Alerts, and Clinical Decision Support. Nephron.

[28] Park S, Baek SH, Ahn S, Lee K, Hwang H, Ryu J, Ahn SY, Chin HJ, Na KY, Chae D, Kim S (2018). Impact of Electronic Acute Kidney Injury (AKI) Alerts With Automated Nephrologist Consultation on Detection and Severity of AKI: A Quality Improvement Study. Am J Kidney Dis.

[29] Sundararajan V, Henderson T, Perry C, Muggivan A, Quan H, Ghali WA (2004). New ICD-10 version of the Charlson comorbidity index predicted in-hospital mortality. J Clin Epidemiol.

[30] KDIGO Work Group (2012). KDIGO clinical practice guideline for acute kidney injury. Kidney Int Suppl.

[31] Lundberg S, Lee S-I (2017). A Unified Approach to Interpreting Model Predictions. https://arxiv.org/pdf/1705.07874.pdf.

[32] da Cruz HF, Pfahringer B, Martensen T, Schneider F, Meyer A, Böttinger E, Schapranow M (2021). Using interpretability approaches to update "black-box" clinical prediction models: an external validation study in nephrology. Artif Intell Med.

[33] Molnar C (2020). Interpretable machine learning: a guide for making black box models explainable.

[34] Friedman JH (2001). Greedy function approximation: A gradient boosting machine. Ann. Statist.

[35] Kashani K, Rosner MH, Haase M, Lewington AJP, O'Donoghue DJ, Wilson FP, Nadim MK, Silver SA, Zarbock A, Ostermann M, Mehta RL, Kane-Gill SL, Ding X, Pickkers P, Bihorac A, Siew ED, Barreto EF, Macedo E, Kellum JA, Palevsky PM, Tolwani AJ, Ronco C, Juncos LA, Rewa OG, Bagshaw SM, Mottes TA, Koyner JL, Liu KD, Forni LG, Heung M, Wu V (2019). Quality Improvement Goals for Acute Kidney Injury. Clin J Am Soc Nephrol.

[36] Wijeysundera DN, Karkouti K, Dupuis J, Rao V, Chan CT, Granton JT, Beattie WS (2007). Derivation and validation of a simplified predictive index for renal replacement therapy after cardiac surgery. JAMA.

[37] Malhotra R, Kashani KB, Macedo E, Kim J, Bouchard J, Wynn S, Li G, Ohno-Machado L, Mehta R (2017). A risk prediction score for acute kidney injury in the intensive care unit. Nephrol Dial Transplant.

[38] Basu RK, Zappitelli M, Brunner L, Wang Y, Wong HR, Chawla LS, Wheeler DS, Goldstein SL (2014). Derivation and validation of the renal angina index to improve the prediction of acute kidney injury in critically ill children. Kidney Int.

[39] Kim DW, Jang HY, Kim KW, Shin Y, Park SH (2019). Design Characteristics of Studies Reporting the Performance of Artificial Intelligence Algorithms for Diagnostic Analysis of Medical Images: Results from Recently Published Papers. Korean J Radiol.

[40] Kate RJ, Perez RM, Mazumdar D, Pasupathy KS, Nilakantan V (2016). Prediction and detection models for acute kidney injury in hospitalized older adults. BMC Med Inform Decis Mak.

[41] Mohamadlou H, Lynn-Palevsky A, Barton C, Chettipally U, Shieh L, Calvert J, Saber NR, Das R (2018). Prediction of Acute Kidney Injury With a Machine Learning Algorithm Using Electronic Health Record Data. Can J Kidney Health Dis.

[42] He J, Hu Y, Zhang X, Wu L, Waitman LR, Liu M (2019). Multi-perspective predictive modeling for acute kidney injury in general hospital populations using electronic medical records. JAMIA Open.

[43] Warnock DG (2017). The pressing need for real-time risk assessment of hospital-acquired acute kidney injury. Nephrol Dial Transplant.

[44] Williams RJ, Zipser D (1989). A Learning Algorithm for Continually Running Fully Recurrent Neural Networks. Neural Computation.

[45] Mikolov T, Kombrink S, Burget L, Cernocký J, Khudanpur S (2011). Extensions of recurrent neural network language model.

[46] Choi E, Schuetz A, Stewart WF, Sun J (2017). Using recurrent neural network models for early detection of heart failure onset. J Am Med Inform Assoc.

[47] Koyner JL, Carey KA, Edelson DP, Churpek MM (2018). The Development of a Machine Learning Inpatient Acute Kidney Injury Prediction Model. Crit Care Med.

[48] Churpek MM, Carey KA, Edelson DP, Singh T, Astor BC, Gilbert ER, Winslow C, Shah N, Afshar M, Koyner JL (2020). Internal and External Validation of a Machine Learning Risk Score for Acute Kidney Injury. JAMA Netw Open.

[49] Song X, Yu ASL, Kellum JA, Waitman LR, Matheny ME, Simpson SQ, Hu Y, Liu M (2020). Cross-site transportability of an explainable artificial intelligence model for acute kidney injury prediction. Nat Commun.

[50] Debray TPA, Vergouwe Y, Koffijberg H, Nieboer D, Steyerberg EW, Moons KGM (2015). A new framework to enhance the interpretation of external validation studies of clinical prediction models. J Clin Epidemiol.

