# Supplementary material for: Real-Time Clinical Decision Support Based on Recurrent Neural Networks for In-Hospital Acute Kidney Injury: External Validation and Model Interpretation
Source: J Med Internet Res. 2021 Apr 16;23(4):e24120. doi: 10.2196/24120 (PMC8087972; doi:10.2196/24120)
Supplement: Multimedia Appendix 1 [file jmir_v23i4e24120_app1.docx]

**Multimedia Appendix 1.** List of variables used in model development.

| Category | Variables | Numbers |
| --- | --- | --- |
| Demographics | Age, sex, BMI, weight, height | 5 |
| Laboratory tests^§^ | Creatinine, WBC, hemoglobin, platelet, albumin, sodium, potassium, chloride, AST, ALT, BUN, total CO_2_, bilirubin, calcium, glucose, CK (3 categories), lipase (3 categories), troponin I (3 categories), and baseline eGFR | 25 |
| Vitals^§^ | Systolic blood pressure, diastolic blood pressure, pulse rate, mean arterial pressure, body temperature (mean, maximum, minimum per day) | 15 |
| Comorbidities | Ischemic heart disease, hypertension, diabetes mellitus, heart failure, liver disease, cerebrovascular disease, chronic obstructive pulmonary disease, chronic kidney disease, previous AKI, cancer, HIV, and CCI | 12 |
| Medications^§^ | NSAIDs, diuretics, acyclovir, aminoglycoside, amphotericin, beta blocker, calcium channel blocker, cisplatin, vancomycin, colistin, cyclosporin, renin-angiotensin blocker, vasopressors, statins (medication use during and before admission) | 28 |
| Clinical conditions | Departments (14 categories), mechanical ventilation (current/recent)^§^, ICU admission, surgery (major/minor)^§^, anesthesia (general/non-general) ^§^, surgery duration^§^ | 22 |
| Abbreviations: BMI, body mass index; WBC, white blood cell; AST, asparate aminotransferase; ALT, alanine aminotransferase; BUN, blood urea nitrogen; CK, creatine kinase; HIV, human immunodeficiency virus; CCI, Charlson comorbidity index; NSAIDs, non-steroidal anti-inflammatory drug; ICU, intensive care unit. ^§^These variables are time-updated dynamic variables except for baseline eGFR. | | |
