# Supplementary figures and images for: Real-Time Clinical Decision Support Based on Recurrent Neural Networks for In-Hospital Acute Kidney Injury: External Validation and Model Interpretation
Source: J Med Internet Res. 2021 Apr 16;23(4):e24120. doi: 10.2196/24120 (PMC8087972; doi:10.2196/24120)

Multimedia Appendix 2. Flow diagram for study participants.


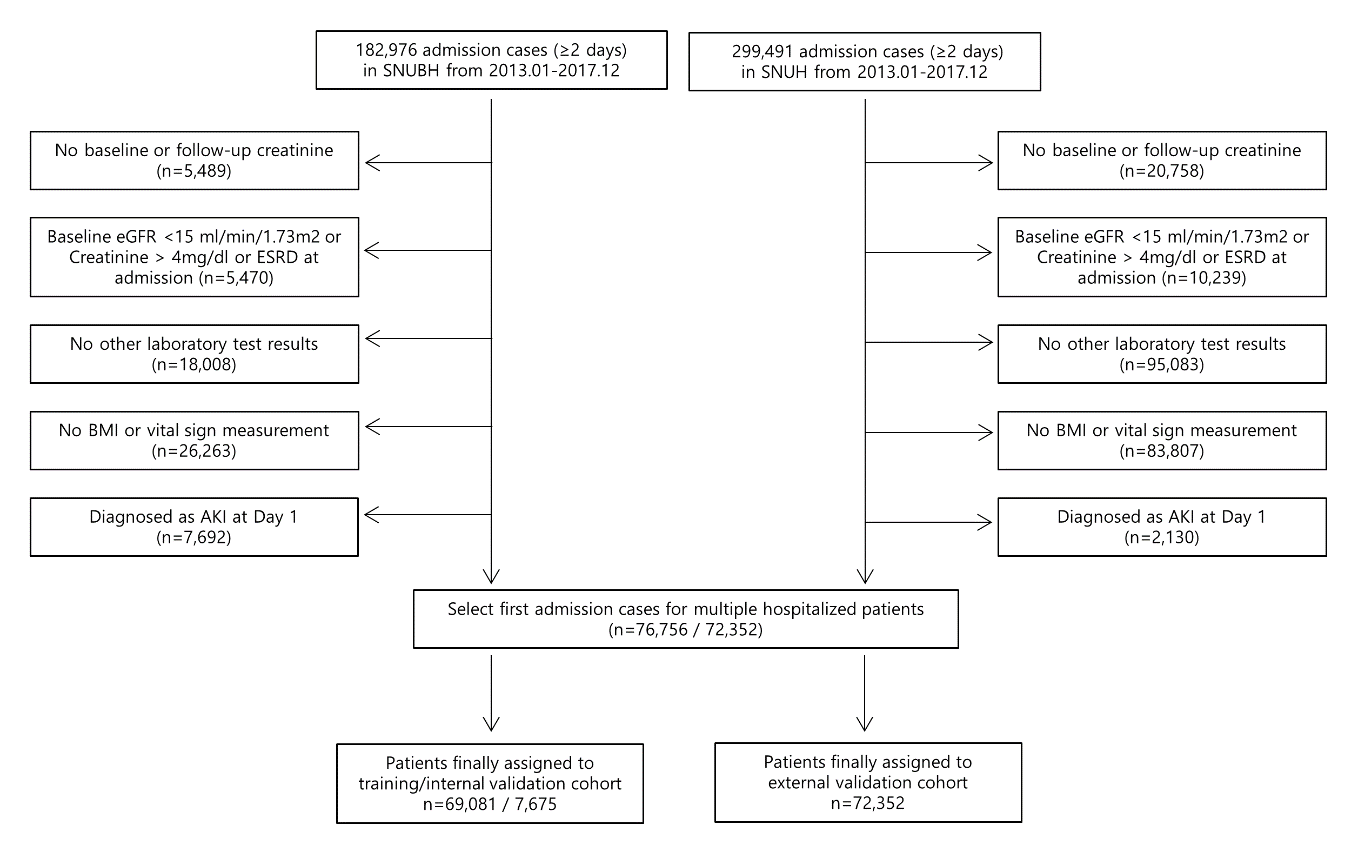

Supplement: Multimedia Appendix 2 [file jmir_v23i4e24120_app2.docx]

**Multimedia Appendix 7.** SHAP feature importance plot for model 1. (A) The RNN model, (B) the XGBoost model.


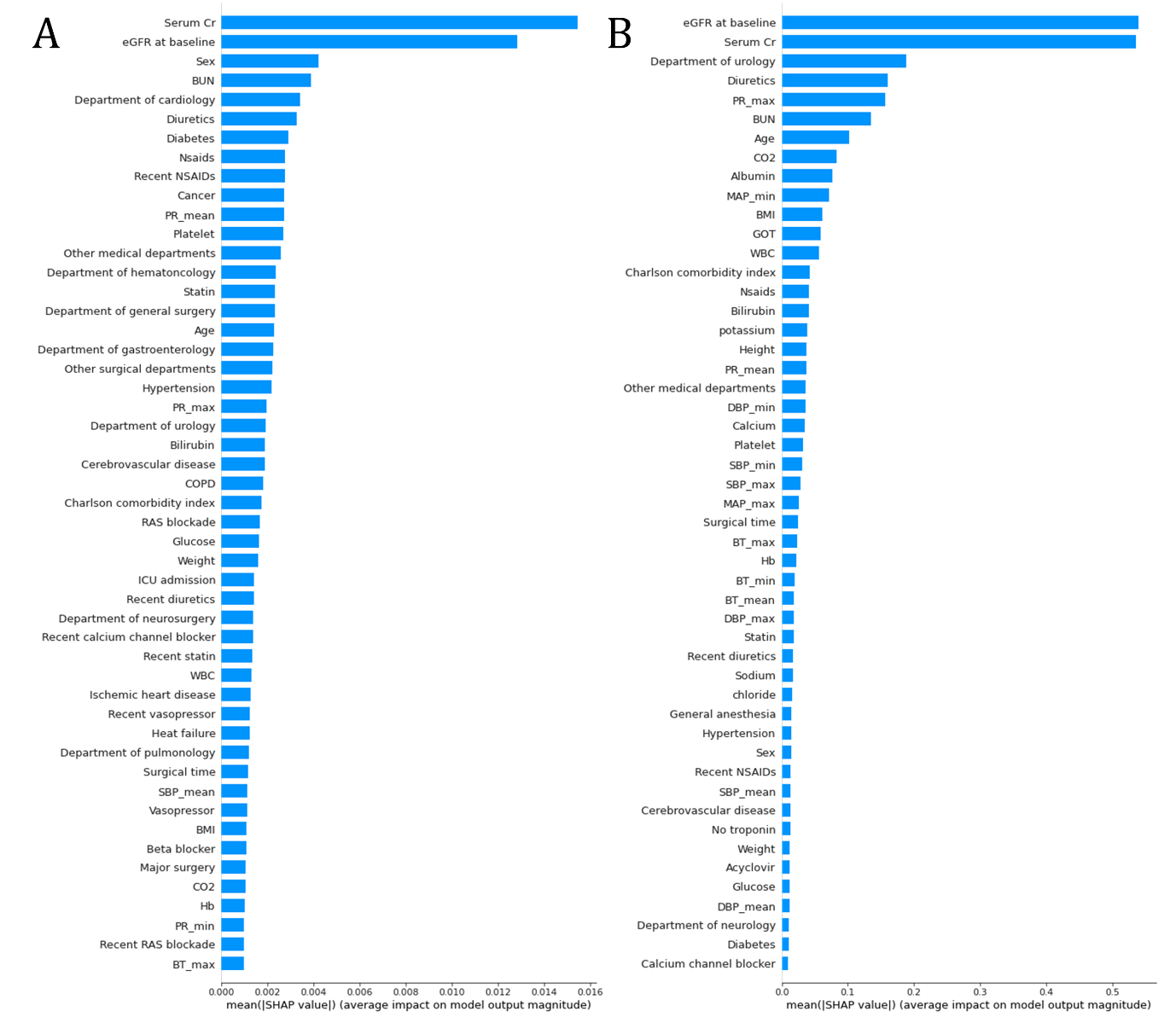

Supplement: Multimedia Appendix 7 [file jmir_v23i4e24120_app7.docx]
