# Supplementary material for: Real-Time Clinical Decision Support Based on Recurrent Neural Networks for In-Hospital Acute Kidney Injury: External Validation and Model Interpretation
Source: J Med Internet Res. 2021 Apr 16;23(4):e24120. doi: 10.2196/24120 (PMC8087972; doi:10.2196/24120)

**Multimedia Appendix 3.** Cumulative incidence of AKI. Red: any AKI in training cohort, green: any AKI in external validation cohort, blue: stage 2 or higher AKI in training cohort, and purple: stage 2 or higher AKI in external validation cohort. AKI diagnosed on the first day of hospitalization was excluded.


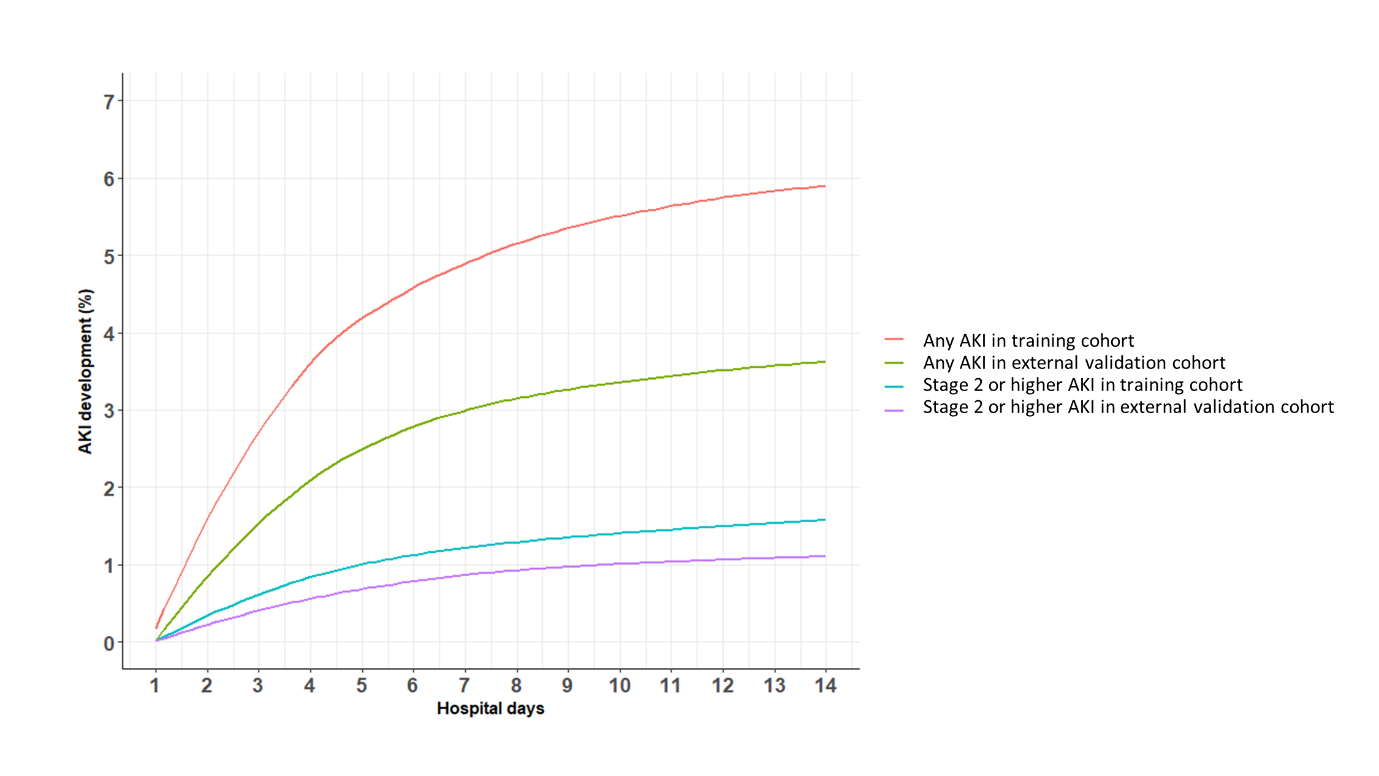

Supplement: Multimedia Appendix 3 [file jmir_v23i4e24120_app3.docx]
