# Supplementary material for: Real-Time Clinical Decision Support Based on Recurrent Neural Networks for In-Hospital Acute Kidney Injury: External Validation and Model Interpretation
Source: J Med Internet Res. 2021 Apr 16;23(4):e24120. doi: 10.2196/24120 (PMC8087972; doi:10.2196/24120)

**Multimedia Appendix 4.** The AUC of the developed model according to different training sample sizes. The percentages indicate the proportion of the training dataset from the data in SNUBH.


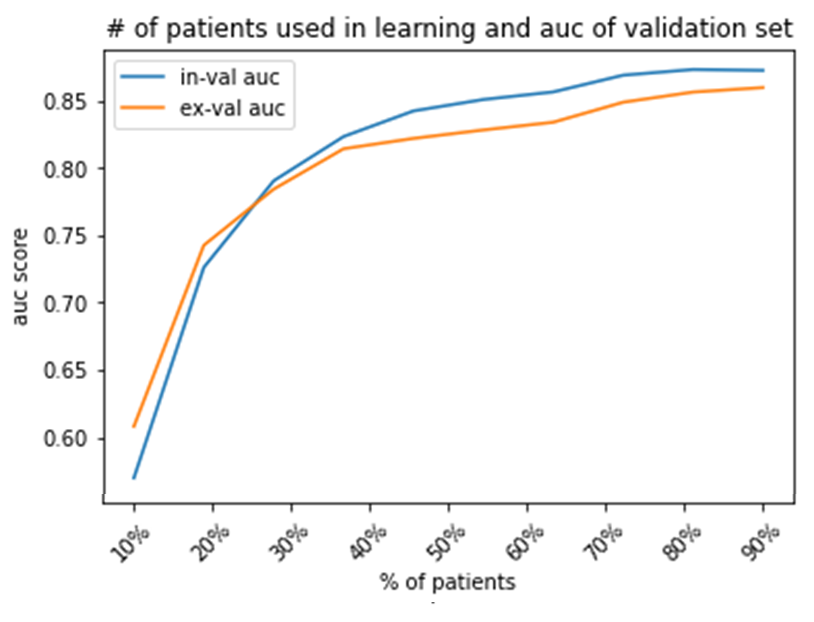

Supplement: Multimedia Appendix 4 [file jmir_v23i4e24120_app4.docx]
