# Supplementary material for: Real-Time Clinical Decision Support Based on Recurrent Neural Networks for In-Hospital Acute Kidney Injury: External Validation and Model Interpretation
Source: J Med Internet Res. 2021 Apr 16;23(4):e24120. doi: 10.2196/24120 (PMC8087972; doi:10.2196/24120)
Supplement: Multimedia Appendix 5 [file jmir_v23i4e24120_app5.docx]

**Multimedia Appendix 5.** Evaluation metrics of model 1 for different probability cutoffs (any stage AKI)

| Cutoffs | Metrics | Stacked RNN | | | XGBoost | | |
| --- | --- | --- | --- | --- | --- | --- | --- |
|  |  | Internal | External | External, updated | Internal | External | External, updated |
| 0.80 | ACC | 0.939 | 0.933 | 0.937 | 0.950 | 0.964 | 0.960 |
|  | Sens | 0.394 | 0.351 | 0.382 | 0.305 | 0.186 | 0.249 |
|  | Spec | 0.956 | 0.954 | 0.955 | 0.972 | 0.985 | 0.979 |
|  | PPV | 0.22 | 0.211 | 0.222 | 0.279 | 0.256 | 0.250 |
|  | NPV | 0.98 | 0.977 | 0.979 | 0.976 | 0.978 | 0.979 |
|  | F1 | 0.282 | 0.246 | 0.281 | 0.291 | 0.216 | 0.250 |
| 0.75 | ACC | 0.921 | 0.914 | 0.918 | 0.937 | 0.954 | 0.950 |
|  | Sens | 0.476 | 0.426 | 0.460 | 0.384 | 0.269 | 0.329 |
|  | Spec | 0.935 | 0.931 | 0.934 | 0.957 | 0.973 | 0.967 |
|  | PPV | 0.187 | 0.178 | 0.189 | 0.237 | 0.216 | 0.215 |
|  | NPV | 0.983 | 0.979 | 0.981 | 0.978 | 0.980 | 0.981 |
|  | F1 | 0.268 | 0.251 | 0.268 | 0.293 | 0.239 | 0.260 |
| 0.70 | ACC | 0.904 | 0.895 | 0.900 | 0.922 | 0.943 | 0.936 |
|  | Sens | 0.576 | 0.514 | 0.555 | 0.475 | 0.342 | 0.410 |
|  | Spec | 0.914 | 0.908 | 0.911 | 0.937 | 0.960 | 0.951 |
|  | PPV | 0.173 | 0.164 | 0.173 | 0.210 | 0.191 | 0.186 |
|  | NPV | 0.986 | 0.982 | 0.984 | 0.981 | 0.981 | 0.983 |
|  | F1 | 0.267 | 0.249 | 0.264 | 0.291 | 0.245 | 0.256 |
| 0.65 | ACC | 0.883 | 0.875 | 0.879 | 0.904 | 0.932 | 0.919 |
|  | Sens | 0.633 | 0.571 | 0.611 | 0.548 | 0.402 | 0.490 |
|  | Spec | 0.891 | 0.885 | 0.888 | 0.917 | 0.946 | 0.931 |
|  | PPV | 0.154 | 0.149 | 0.155 | 0.187 | 0.171 | 0.163 |
|  | NPV | 0.987 | 0.983 | 0.986 | 0.983 | 0.983 | 0.985 |
|  | F1 | 0.248 | 0.236 | 0.247 | 0.279 | 0.240 | 0.245 |
| 0.60 | ACC | 0.862 | 0.854 | 0.858 | 0.882 | 0.918 | 0.899 |
|  | Sens | 0.69 | 0.625 | 0.667 | 0.596 | 0.462 | 0.562 |
|  | Spec | 0.867 | 0.862 | 0.864 | 0.892 | 0.930 | 0.908 |
|  | PPV | 0.141 | 0.137 | 0.142 | 0.162 | 0.155 | 0.144 |
|  | NPV | 0.989 | 0.985 | 0.967 | 0.984 | 0.984 | 0.987 |
|  | F1 | 0.234 | 0.225 | 0.234 | 0.254 | 0.232 | 0.229 |
| 0.55 | ACC | 0.842 | 0.834 | 0.838 | 0.856 | 0.901 | 0.873 |
|  | Sens | 0.752 | 0.679 | 0.722 | 0.661 | 0.517 | 0.634 |
|  | Spec | 0.845 | 0.839 | 0.842 | 0.863 | 0.912 | 0.880 |
|  | PPV | 0.132 | 0.129 | 0.133 | 0.144 | 0.139 | 0.127 |
|  | NPV | 0.991 | 0.987 | 0.989 | 0.986 | 0.956 | 0.989 |
|  | F1 | 0.225 | 0.217 | 0.225 | 0.237 | 0.219 | 0.211 |
| 0.50 | ACC | 0.820 | 0.812 | 0.817 | 0.827 | 0.880 | 0.845 |
|  | Sens | 0.800 | 0.724 | 0.772 | 0.716 | 0.572 | 0.690 |
|  | Spec | 0.820 | 0.815 | 0.818 | 0.831 | 0.889 | 0.850 |
|  | PPV | 0.123 | 0.121 | 0.125 | 0.129 | 0.125 | 0.112 |
|  | NPV | 0.992 | 0.988 | 0.991 | 0.988 | 0.987 | 0.990 |
|  | F1 | 0.213 | 0.207 | 0.215 | 0.219 | 0.205 | 0.193 |

^a^PPV, positive predictive value; ^b^NPV, negative predictive value; ^c^F1, F1-score; ^d^AUC, the area under the ROC curve.
