# Supplementary material for: Real-Time Clinical Decision Support Based on Recurrent Neural Networks for In-Hospital Acute Kidney Injury: External Validation and Model Interpretation
Source: J Med Internet Res. 2021 Apr 16;23(4):e24120. doi: 10.2196/24120 (PMC8087972; doi:10.2196/24120)
Supplement: Multimedia Appendix 6 [file jmir_v23i4e24120_app6.docx]

**Multimedia Appendix 6.** Evaluation metrics of model 1 for different probability cutoffs (AKI stage ≥2)

| Cutoffs | Metrics | Stacked RNN | | | XGBoost | | |
| --- | --- | --- | --- | --- | --- | --- | --- |
|  |  | Internal | External | External, updated | Internal | External | External, updated |
| 0.90 | ACC | 0.981 | 0.98 | 0.98 | 0.984 | 0.988 | 0.980 |
|  | Sens | 0.452 | 0.394 | 0.414 | 0.284 | 0.193 | 0.356 |
|  | Spec | 0.984 | 0.984 | 0.984 | 0.991 | 0.995 | 0.986 |
|  | PPV | 0.14 | 0.155 | 0.152 | 0.233 | 0.269 | 0.180 |
|  | NPV | 0.997 | 0.995 | 0.996 | 0.993 | 0.993 | 0.994 |
|  | F1 | 0.213 | 0.222 | 0.222 | 0.256 | 0.225 | 0.239 |
| 0.85 | ACC | 0.973 | 0.971 | 0.972 | 0.977 | 0.985 | 0.971 |
|  | Sens | 0.563 | 0.474 | 0.505 | 0.411 | 0.283 | 0.432 |
|  | Spec | 0.975 | 0.975 | 0.975 | 0.983 | 0.991 | 0.975 |
|  | PPV | 0.115 | 0.124 | 0.121 | 0.191 | 0.216 | 0.133 |
|  | NPV | 0.997 | 0.996 | 0.997 | 0.994 | 0.994 | 0.995 |
|  | F1 | 0.191 | 0.196 | 0.195 | 0.261 | 0.245 | 0.204 |
| 0.80 | ACC | 0.963 | 0.962 | 0.962 | 0.967 | 0.980 | 0.962 |
|  | Sens | 0.615 | 0.516 | 0.538 | 0.506 | 0.338 | 0.495 |
|  | Spec | 0.965 | 0.965 | 0.965 | 0.972 | 0.986 | 0.967 |
|  | PPV | 0.093 | 0.099 | 0.095 | 0.151 | 0.170 | 0.115 |
|  | NPV | 0.998 | 0.996 | 0.997 | 0.995 | 0.994 | 0.995 |
|  | F1 | 0.162 | 0.166 | 0.161 | 0.232 | 0.227 | 0.187 |
| 0.75 | ACC | 0.955 | 0.952 | 0.953 | 0.958 | 0.974 | 0.950 |
|  | Sens | 0.69 | 0.58 | 0.60 | 0.558 | 0.404 | 0.563 |
|  | Spec | 0.956 | 0.955 | 0.955 | 0.961 | 0.979 | 0.953 |
|  | PPV | 0.084 | 0.087 | 0.084 | 0.125 | 0.147 | 0.097 |
|  | NPV | 0.998 | 0.997 | 0.997 | 0.995 | 0.995 | 0.996 |
|  | F1 | 0.149 | 0.152 | 0.152 | 0.205 | 0.216 | 0.165 |
| 0.70 | ACC | 0.943 | 0.941 | 0.942 | 0.947 | 0.968 | 0.941 |
|  | Sens | 0.741 | 0.641 | 0.662 | 0.638 | 0.451 | 0.621 |
|  | Spec | 0.945 | 0.943 | 0.994 | 0.950 | 0.973 | 0.944 |
|  | PPV | 0.072 | 0.077 | 0.074 | 0.112 | 0.126 | 0.088 |
|  | NPV | 0.998 | 0.997 | 0.998 | 0.996 | 0.995 | 0.996 |
|  | F1 | 0.131 | 0.138 | 0.133 | 0.191 | 0.198 | 0.155 |
| 0.65 | ACC | 0.932 | 0.928 | 0.930 | 0.936 | 0.960 | 0.926 |
|  | Sens | 0.763 | 0.666 | 0.690 | 0.682 | 0.504 | 0.656 |
|  | Spec | 0.933 | 0.930 | 0.932 | 0.938 | 0.964 | 0.928 |
|  | PPV | 0.062 | 0.066 | 0.064 | 0.099 | 0.110 | 0.075 |
|  | NPV | 0.999 | 0.997 | 0.998 | 0.997 | 0.995 | 0.997 |
|  | F1 | 0.115 | 0.121 | 0.118 | 0.172 | 0.181 | 0.134 |
| 0.60 | ACC | 0.919 | 0.915 | 0.917 | 0.922 | 0.951 | 0.914 |
|  | Sens | 0.80 | 0.70 | 0.73 | 0.729 | 0.552 | 0.698 |
|  | Spec | 0.92 | 0.917 | 0.918 | 0.924 | 0.954 | 0.916 |
|  | PPV | 0.055 | 0.059 | 0.057 | 0.087 | 0.095 | 0.068 |
|  | NPV | 0.999 | 0.998 | 0.998 | 0.997 | 0.996 | 0.997 |
|  | F1 | 0.102 | 0.108 | 0.106 | 0.115 | 0.163 | 0.124 |

^a^PPV, positive predictive value; ^b^NPV, negative predictive value; ^c^F1, F1-score; ^d^AUC, the area under the ROC curve.
