# Supplementary material for: Real-Time Clinical Decision Support Based on Recurrent Neural Networks for In-Hospital Acute Kidney Injury: External Validation and Model Interpretation
Source: J Med Internet Res. 2021 Apr 16;23(4):e24120. doi: 10.2196/24120 (PMC8087972; doi:10.2196/24120)

**Multimedia Appendix 8.** SHAP feature importance plots according to different time points. From (A) 3 days before the prediction time, (B) 2 days before the prediction time, and (C) 1 day before the prediction time.


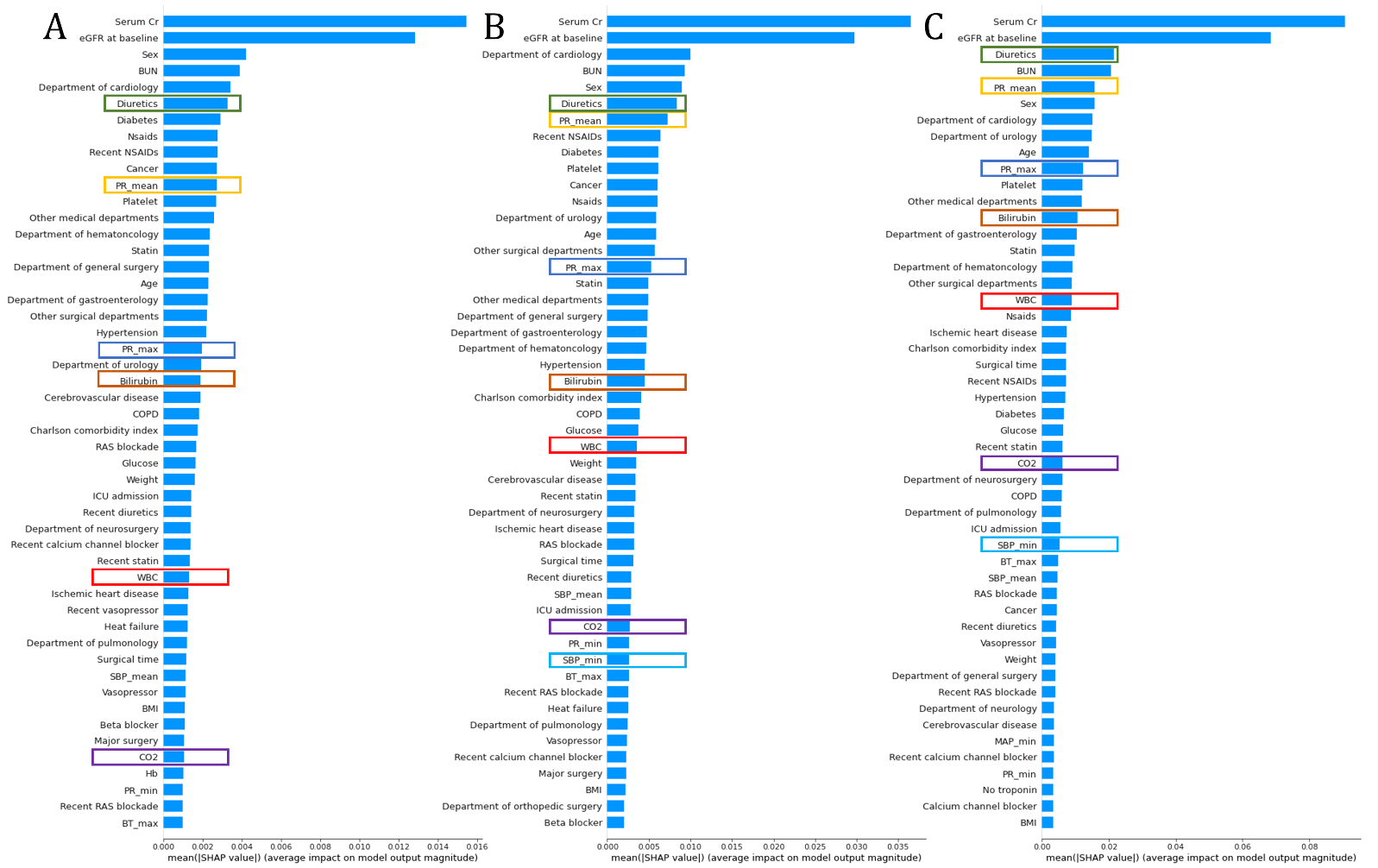

Supplement: Multimedia Appendix 8 [file jmir_v23i4e24120_app8.docx]
