# Supplementary material for: Real-Time Clinical Decision Support Based on Recurrent Neural Networks for In-Hospital Acute Kidney Injury: External Validation and Model Interpretation
Source: J Med Internet Res. 2021 Apr 16;23(4):e24120. doi: 10.2196/24120 (PMC8087972; doi:10.2196/24120)

**Multimedia Appendix 9.** Partial dependence plots and individual conditional expectation plots. ICE plots for 100 randomly selected patients are shown. Hb, hemoglobin; WBC, white blood cell count; AST, aspartate aminotransferase; ALT, alanine aminotransferase; SBP, systolic blood pressure; DBP, diastolic blood pressure; MAP, mean arterial blood pressure; PR, pulse rate; BT, body temperature; Max, maximum; Min, minimum.


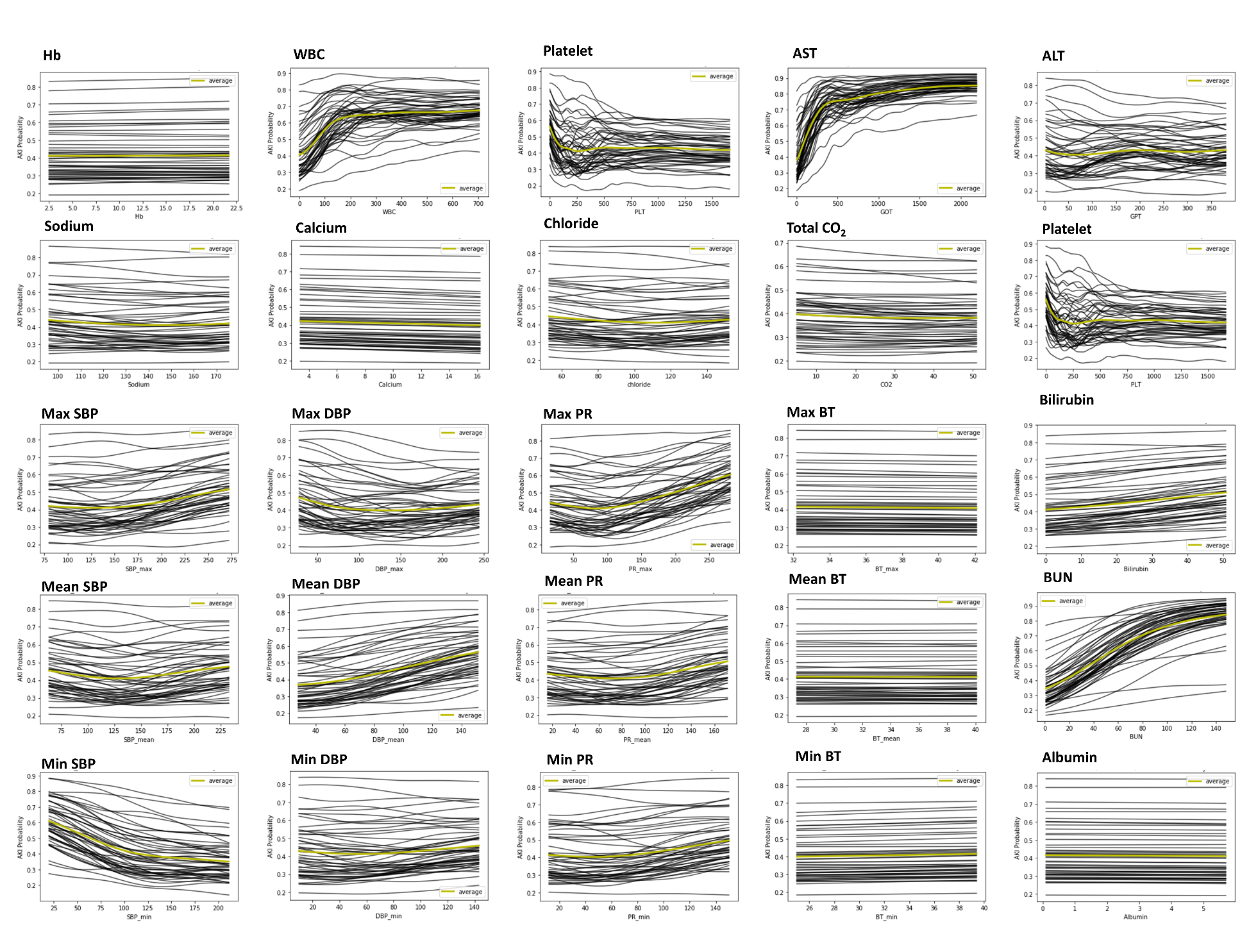

Supplement: Multimedia Appendix 9 [file jmir_v23i4e24120_app9.docx]
